# Prenatal exposure to a low dose of BPS causes sex-dependent alterations to vascular endothelial function in adult offspring

**DOI:** 10.3389/ftox.2022.933572

**Published:** 2022-10-13

**Authors:** Liam T. Connors, Hai-Lei Zhu, Manvir Gill, Emma Walsh, Radha D. Singh, Sarah Easson, Sofia B. Ahmed, Hamid R. Habibi, William C. Cole, Jennifer A. Thompson

**Affiliations:** ^1^ Department of Physiology and Pharmacology, University of Calgary, Calgary, AB, Canada; ^2^ Libin Cardiovascular Institute, University of Calgary, Calgary, AB, Canada; ^3^ Alberta Children’s Hospital Research Institute, University of Calgary, Calgary, AB, Canada; ^4^ Cumming School of Medicine, University of Calgary, Calgary, AB, Canada; ^5^ Department of Biological Sciences, University of Calgary, Calgary, AB, Canada

**Keywords:** Endothelium, nitric oxide, estrogen, development, microvascular, bisphenol S (BPS)

## Abstract

**Background:** Bisphenol S (BPS) is among the most commonly used substitutes for Bisphenol A (BPA), an endocrine disrupting chemical used as a plasticizer in the manufacture of polycarbonate plastics and epoxy resins. Bisphenols interfere with estrogen receptor (ER) signaling, which modulates vascular function through stimulation of nitric oxide (NO) production *via* endothelial nitric oxide synthase (eNOS). BPS can cross into the placenta and accumulates in the fetal compartment to a greater extent than BPA, potentially interfering with key developmental events. Little is known regarding the developmental impact of exposure to BPA substitutes, particularly with respect to the vasculature.

**Objective:** To determine if prenatal BPS exposure influences vascular health in adulthood.

**Methods:** At the time of mating, female C57BL/6 dams were administered BPS (250 nM) or vehicle control in the drinking water, and exposure continued during lactation. At 12-week of age, mesenteric arteries were excised from male and female offspring and assessed for responses to an endothelium-dependent (acetylcholine, ACh) and endothelium-independent (sodium nitroprusside, SNP) vasodilator. Endothelium-dependent dilation was measured in the presence or absence of L-NAME, an eNOS inhibitor. To further explore the role of NO and ER signaling, wire myography was used to assess ACh responses in aortic rings after acute exposure to BPS in the presence or absence of L-NAME or an ER antagonist.

**Results:** Increased ACh dilation and increased sensitivity to Phe were observed in microvessels from BPS-exposed females, while no changes were observed in male offspring. Differences in ACh-induced dilation between control or BPS-exposed females were eliminated with L-NAME. Increased dilatory responses to ACh after acute BPS exposure were observed in aortic rings from female mice only, and differences were eliminated with inhibition of eNOS or inhibition of ER.

**Conclusion:** Prenatal BPS exposure leads to persistent changes in endothelium-dependent vascular function in a sex-specific manner that appears to be modulated by interaction of BPS with ER signaling.

## Introduction

Bisphenols, used as plasticizers in the manufacture of polycarbonate plastics and epoxy resins, are among the world’s most ubiquitous chemical pollutants (Ribeiro et al., 2017). The plastic industry has increasingly replaced the most common bisphenol, BPA, with structural analogues such as BPS and BPF, after regulatory bans on the use and import of BPA-containing infant products introduced by several countries in the late 2000s. In some countries, exposure levels of BPA substitutes are approaching that of BPA. For instance, BPS has been detected in 81% of urine samples collected from adults in the United States and Asia (Liao et al., 2012). Bisphenols are designated as endocrine disrupting chemicals (EDC) that interfere with endocrine signaling by mimicking endogenous ligands and act as agonists or antagonists depending on the dose and cell-type. BPA is known to interact with estrogen receptors, ERα and ERβ, albeit at a ∼1000-to-2000-fold lower affinity than endogenous estrogen (Viñas and Watson, 2013). Although new substitutes for BPA remain understudied, existing data reveal BPS to exhibit estrogenic properties similar to its predecessor (Park et al., 2020).

Bisphenols have been detected in breast milk, amniotic fluid, and fetal tissues (Lee et al., 2018), and thus exposure occurs during critical developmental windows in early life that are particularly sensitive to endocrine disruption. Toxicokinetic studies have shown that due to immature detoxification defences in the fetus, the half-life of bisphenols is 20-fold higher in the fetal compartment compared to the adult circulation (Braun et al., 2011; Grandin et al., 2018; Silva et al., 2019). Further, a study conducted in pregnant sheep showed that while BPS crosses the placenta with lower efficiency than BPA, it accumulates in the fetal compartment to a greater extent due to its slower clearance (Gingrich et al., 2019). Thus, the fetus is exposed to BPA and its analogues through maternal intake and there is evidence showing that *in utero* exposure interferes with developmental processes and programs persistent changes in organ function (Shi et al., 2019; Ahn et al., 2020; Zhou et al., 2020).

Estrogen receptors are expressed in both vascular endothelial and smooth muscle cells and are thought to play a major role in mediating sex differences in vascular function. In endothelial cells, ERα localizes to caveolae in the cell membrane where it activates Akt-dependent phosphorylation of eNOS and NO-dependent vasodilation upon binding to estrogen (Haynes et al., 2000; Knowlton and Lee, 2012). ERα or ERβ localized in the cytoplasm or nucleus of endothelial cells dimerize and bind to estrogen response elements (EREs), leading to the recruitment of transcription factors to induce the transcription of eNOS (Chambliss et al., 2002; Wu et al., 2011; Yaşar et al., 2017). Since estrogen receptors modulate eNOS expression and NO production, it is possible that exposure to BPA or its analogues influence vascular function. Although few studies have investigated the impact of bisphenol exposure on the cardiovascular system, there is some evidence to suggest that exposure to BPA in the postnatal period influences blood pressure and endothelial cell function (Saura et al., 2014; Sanchez-esteban et al., 2020). The current study aimed to determine how gestational exposure to the new BPA substitute, BPS, influences later-life vascular function.

## Materials and methods

### Animal model

At the time of mating, 12-week-old virgin C57BL6 female mice housed in cages with no plastic enrichment were provided with a phytoestrogen-low diet (Envigo) and exposed to BPS (250 nM) or vehicle control (0.01% DMSO) through glass water bottles. Litters were culled to a maximum of six pups and exposure continued throughout lactation until postnatal day 21 (Pd21), at which time the pups were weaned, separated by sex, and transferred to plastic-reduced environment cages equipped with glass water bottles. At 12 weeks of age, offspring were anesthetized and sacrificed by decapitation for collection of mesenteric arteries. While there are no currently established tolerable daily intake (TDI) limits for BPA analogues, the current TDI for BPA was used to guide the dose of BPS used in the current study. A concentration of 250 nM of BPS in the drinking water reflects a human equivalent estimated daily intake (EI_24_) of approximately 8 nmol BPS/kg body weight/day, below the current TDIs for BPA set at 18 nmol BPA/kg body weight/day recommended by the European Food Safety Authority (EFSA) and 219 nmol BPA/kg body weight/day by the Food and Drug Administration (FDA) (Aungst et al., 2014; EFSA Panel on Food Contact Materials et al., 2015). Animals were housed in the University of Calgary Clara Christie Centre for Mouse Genomics facility and treated in accordance with animal ethics protocol AC19-0006 approved by the University of Calgary Animal Care Committee. All animal procedures were carried out in accordance with Canadian council on Animal Care guidelines.

### Development of sexual characteristics

At Pd21, offspring were briefly anesthetized and the distance from the genital base to the anus was recorded to calculate the anogenital distance (AGD). Crown-to-rump length (CRL) and body weight were recorded.

### Pressure myography

At sacrifice, the mesentery was placed in an ice-cold Krebs solution (0.0025 M K^+^ Krebs: 0.12 M NaCl, 0.025 M NaHCO_3_, 0.0048 M KCl, 0.011 M glucose, 2.70E-5 M EDTA, 0.0012 M NaH_2_PO_4_, 0.0012 M of MgSO_4_, and 0.0025 M CaCl_2_; pH 7.4). Fourth order mesenteric arteries were isolated from the surrounding fat and connective tissue and secured onto two diametrically opposed glass cannulas in a pressure myograph chamber (Living Systems Instrumentation) under a dissecting microscope. The bathing chamber was then placed onto the stage of a microscope (Nikon) equipped with live video recording and the proximal end of the vessel was connected to a pressure device (Living Systems Instrumentation) connected to a pressure transducer, while the distal end was closed to maintain stable pressure. The bathing reservoir was filled with the Krebs solution, aerated with a mix of 5.0% CO_2_/air and heated to 37°C. The vessel diameter was recorded using IonOptix software. The intraluminal pressure within the vessels was increased stepwise from 10 to 100 mmHg to observe the development of myogenic tone. Then, the pressure was maintained at 60 mmHg and the vessels were perfused with 0.060 M K^+^ Krebs solution to ascertain contractile viability. The vessels were deemed viable if a contraction greater than or equal to 30 µm was achieved in response to 0.060 M K^+^ Krebs solution. Vessels were then washed with Krebs solution and incubated with increasing concentrations of phenylephrine (Phe: 10^−10^ M–10^−4^ M), a selective α1-adrenergic receptor agonist. Vessels were pre-constricted with 10^−6^ M of Phe before exposure to increasing concentrations of acetylcholine (ACh: 10^−11^ M to 10^−5^ M), an endothelium-dependent vasodilator. A subset of vessels was incubated in 100 µM of the NO synthase inhibitor, NG-nitro-L-arginine methyl ester (L-NAME), prior to exposure to ACh. A single dose of sodium nitroprusside (SNP, 10^−5^ M), an NO donor and endothelium-independent vasodilator, was added to the bath after pre-constriction with 10^−6^ M Phe. After generation of concentration-response curves, vessels were equilibrated with Ca^2+^ free Krebs solution. The pressure was increased stepwise from 10 to 100 mmHg to evaluate the passive response of the vessel to pressure in the absence of myogenic tone. For calculation of % dilation or contraction, diameters were normalized to diameter in Ca^2+^-free Krebs. Passive distensibility was calculated as (D_i_—D_i @ 10 mmHg_)/(D_i @ 10 mmHg_) x 100, where D_i_ = internal diameter. Circumferential wall stress (σ) was calculated as (*p* ⋅ D_i_)/(2⋅WT), where *p* = pressure and WT = wall thickness. Circumferential wall strain (ε) was calculated as (D_i_—D_i @ 10 mmHg_)/D_i @ 10 mmHg._

### Wire myography

Thoracic aortae of C57BL6 male and female mice were isolated and collected in ice cold 2.5 mM K^+^ Krebs solution, stripped of perivascular fat and cut into 3 mm rings that were mounted on two wires in a chamber of a myograph system (DMT). The wire myograph system allowed for evaluation of vasoactive responses in aortic rings with the endothelial layer exposed directly to BPS in the bath. The vessels were equilibrated at a tension of 1.0 g for 45 min in 2.5 mM K^+^ Krebs aerated and warmed to 37 C. The response of the vessel to 1 µM ACh was assessed to determine dilatory function prior to BPS exposure. After washing and equilibration, aortic rings were then incubated in 250 nM BPS for 30 min, after which concentration responses to ACh after pre-contraction were generated. In a subset of vessels, acute exposure to BPS occurred in the presence or absence of 100 μM L-NAME or 1 µM ICI 182,780, to assess the role of eNOS and ER, respectively. The vessels were also exposed to a dose of SNP (100 µM) to evaluate NO-dependent dilation as a positive control for endothelium independent relaxation.

### qRT-PCR

Mesenteric arteries were collected and RNA extracted using the RNeasy^®^ Micro Kit (Qiagen) according to the manufacturer’s protocol. qRT-PCR analysis was carried out using the *Power* SYBR^®^ Green RNA-to-CT™ 1-Step Kit (Applied Biosystems) using 0.1–1 ng RNA. Samples were held at 48°C for 30 min then 95°C for 10 min. Samples were then cycled 40 times at 95°C for 15 s and 58.9°C for 1 min. A melt curve was also performed at 95°C for 15 s, 58.9°C for 15 s, then 95°C for 15 s. Samples were assessed for the targets *eNOS* (Forward: 5′-GGA​CAT​TTT​CGG​ACT​CAC​AT-3′, Reverse: 5′-GCT​GTA​GGC​ATT​CTT​CAG​AG-3′), *ER-α* (Forward: 5′-GAG​AGA​CTG​TCC​AGC​AGT​AA-3′, Reverse: 5′-TGT​GTC​CTT​GAA​TGC​TTC​TC-3′), *ER-β* (Forward: 5′-GTA​ACA​AGG​GCA​TGG​AAC​AT-3′, Reverse: 5′- CCC​ACT​TCT​GAC​CAT​CAT​TG-3′), G-protein coupled estrogen receptor (*GPER*) (Forward: 5′-CAC​CCT​TCT​GGT​TTT​CTG​AG-3′, Reverse: 5′- TTC​AGG​ATT​TGC​TGA​AAG​GG-3′), muscarinic acetylcholine receptor (*mAChR*) (Forward: 5′-CCA​GAT​ATG​ACC​AGC​AAT​GG-3′, Reverse: 5′- GAG​AGT​CTC​TGT​GGT​GTG​TA-3′), and α-1-adrenergic receptor (*α1-AR*) (Forward: 5′-AAT​TTA​CTG​TGC​CTC​ACC​AC-3′, Reverse: 5′- GAG​GGT​GTT​CAA​AGA​AGT​CC-3′), while β-actin (Forward: 5′-3′GAT​CAA​GAT​CAT​TGC​TCC​TCC​T -3′, Reverse: 5′- GTA​ACA​GTC​CGC​CTA​GAA​GC-3′) was used as a housekeeping control.

### Insulin sensitivity and glucose tolerance tests

After a 6-h fast, insulin sensitivity was assessed by injecting 0.5 IU/kg body weight insulin intraperitoneally and sampling blood glucose from the tail at baseline and at 15, 30, 45, 60, and 90-min post-injection. After a recovery period of 4 days, glucose tolerance was assessed by injecting 2 g/kg body weight glucose intraperitoneally and sampling blood glucose from the tail at baseline at 15, 30, 45, 60, and 90-min post-injection.

### Statistical analysis

Statistical analyses were carried out using GraphPad Prism version 9.3.1 for Windows. Two-way ANOVA was performed to assess differences in the E_MAX_ of vessel contraction or dilation. For calculation of the EC_50_ data, concentrations were converted to log form, normalized to maximum response, and EC_50_ was calculated using a curve fit nonlinear regression to compare EC_50_ values. Differences in concentration response curves were also assessed by Two-way ANOVA with Sidak’s multiple comparison test. Students *t*-test was used to assess differences between control and BPS-exposed groups, while two-way ANOVA was used to determine the impact of treatment (BPS) or sex on outcomes. Differences were considered significant if *p* ≤ 0.05 and data are presented as mean ± SEM. For *in vivo* experiments, one male and female offspring per litter was included in each group (BPS or control) for analysis.

## Results

### Anogenital distance is shortened in male pups exposed to a low dose of BPS *in utero*

**FIGURE 1:**
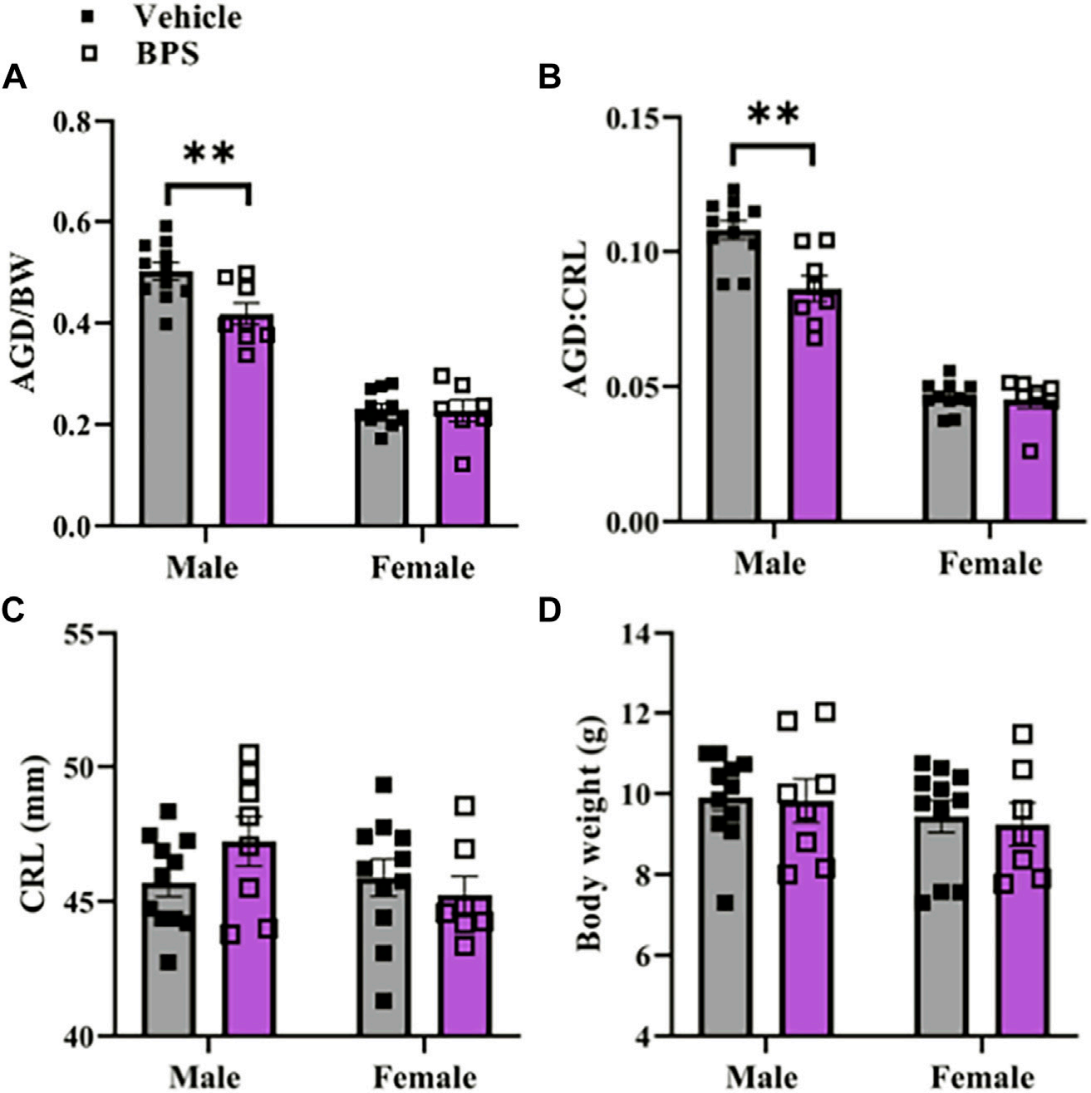
Stunted masculinization in male pups exposed to BPS. Anogenital distance normalized to **(A)** body weight or **(B)** crown-to-rump-length (CRL) in Pd21 male and female mice prenatally exposed to vehicle or BPS. **(C)** Body weight and **(D)** CRL of male and female Pd21 mice. Each sample represents the average of the litter and differences were assessed by Student’s t-test. **, *p* < 0.01 BPS vs. control.

The anogenital distance index, a biomarker of androgen exposure during the *in-utero* window of masculinisation (Liu et al., 2014; Foster et al., 2006; Golub et al., 2010; Swan et al., 2015), was significantly shortened in Pd21 males prenatally exposed to BPS, when normalized to either body weight or CRL (Figures 1A,B). There were no differences in either CRL or body weight in either males or females at Pd21 (Figures 1C,D). Litter size (Supplementary Figure S1A) and sex ratio of the litter (Supplementary Figure S1B) were also unaffected by BPS exposure.

### Prenatal BPS exposure programs sex-dependent changes in microvascular endothelial function

**FIGURE 2:**
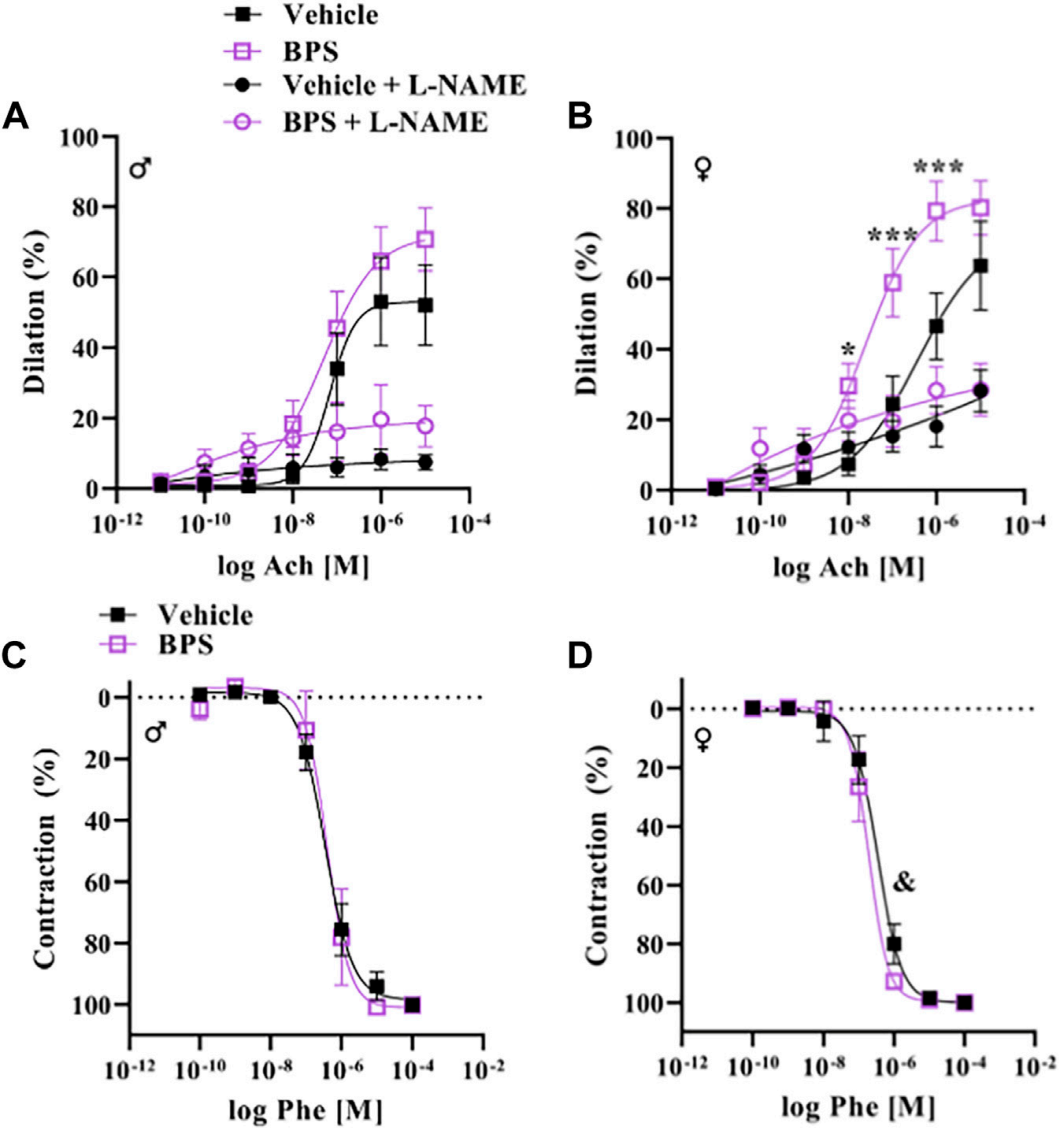
Endothelium-dependent dilation is enhanced in microvessels from BPS-exposed females. Pressure myography was used to assess function in intact 4th order mesenteric arteries isolated from adult male or female mice prenatally exposed to vehicle or BPS. Dilation in response to cumulative concentrations of the endothelium-dependent dilator, acetylcholine (ACh), was calculated as the % internal diameter in relation to pre-constriction diameter, normalized to passive diameter at 60 mmHg. **(A)** ACh-induced dilation in the presence or absence of L-NAME in arteries isolated from male or **(B)** female offspring born to dams exposed to vehicle or BPS. Contractile responses to cumulative concentrations of Phe were calculated as the % of maximum contraction, normalized to passive diameter. **(C)** Phe concentration-response curves of arteries isolated from male and **(D)** female offspring exposed to vehicle or BPS in early life. Dilation at individual concentrations were compared by Two-way ANOVA, followed by the Sidak’s post hoc test. *p < 0.05; ***, *p* < 0.001 BPS vs. control. The EC_50_ was calculated and compared after normalization and fitting to a non-linear curve by the least square method. & *p* < 0.05 of EC_50_ in BPS vs. vehicle control.

**FIGURE 3:**
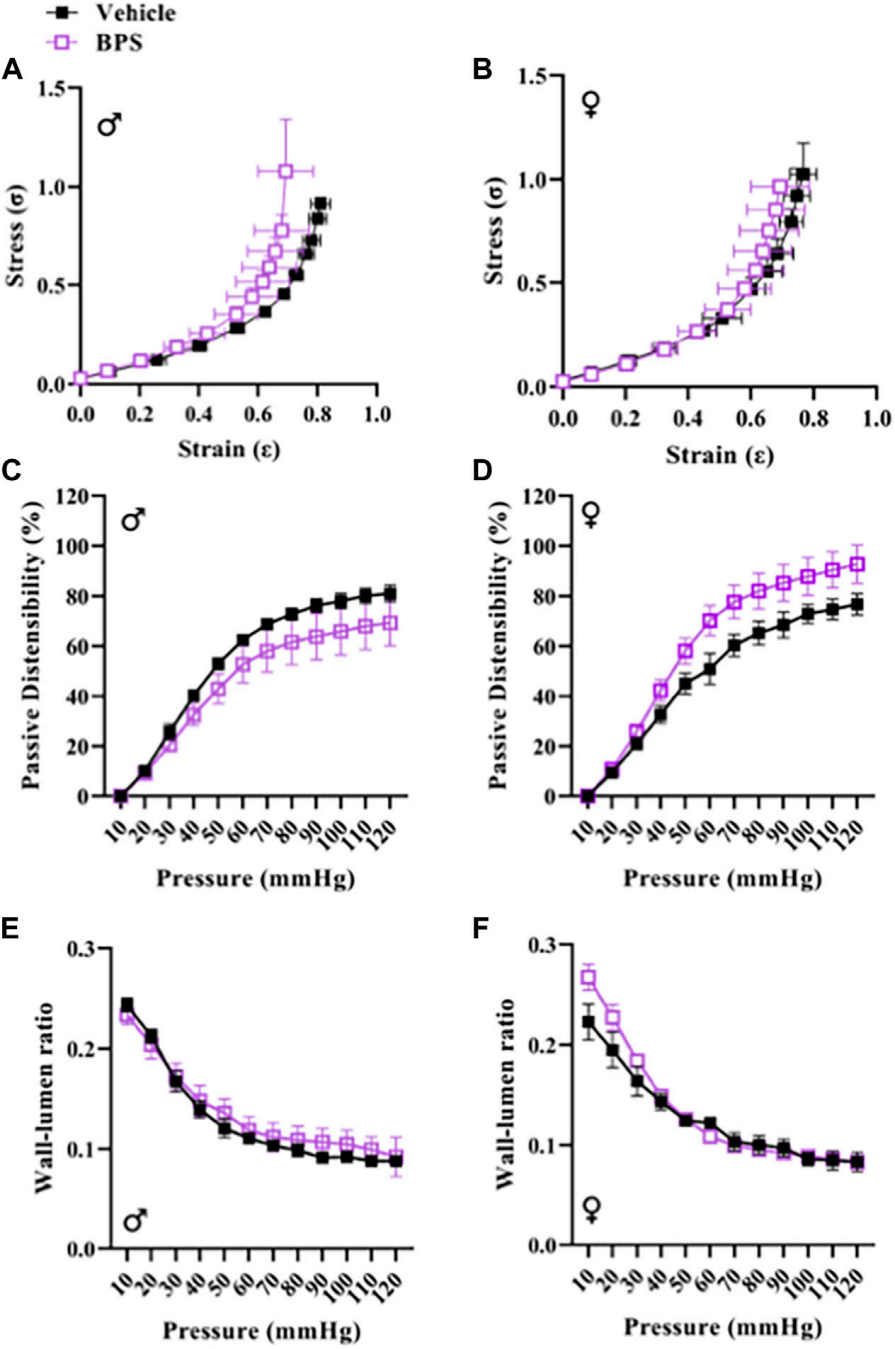
BPS exposure has no impact on passive mechanical function of mesenteric arteries. The stress-strain relationship of 4th order mesenteric arteries isolated from **(A)** male (*n* = 5 control; *n* = 5 BPS) or **(B)** female (*n* = 5 control; *n* = 7 BPS) mice prenatally exposed to BPS. Passive distensibility in mesenteric arteries from **(C)** male or **(D)** female offspring. Wall-lumen ratio in **(E)** male or **(F)** female mesenteric arteries subjected to increasing intraluminal pressure.

While prenatal BPS exposure had no impact on microvascular endothelial function in adult male offspring (Figure 2A), mesenteric arteries isolated from BPS-exposed female offspring exhibited increased Ach-induced vasodilation compared to control females (Figure 2B). The EC_50_ of the dilatory response was also significantly lowered in vessels isolated from BPS-exposed females, indicating increased sensitivity to ACh (Table 1). The difference in ACh-induced dilation between control and BPS-exposed female offspring was abolished by pre-incubation of the vessel with L-NAME (Figure 2B), suggesting that the enhanced dilation is NO-dependent. There were no differences in the E_MAX_ or EC_50_ of contractile responses to Phe in microvessels isolated from male offspring (Figure 2C; Table 1). In microvessels from female offspring, BPS had no impact on the E_MAX_ of contractile responses to Phe; however, the EC_50_ of the Phe response was significantly decreased in BPS-exposed females, indicating an increased sensitivity to Phe (Figure 2D; Table 1). Dilation in response to the NO donor, SNP, was not changed by prenatal BPS exposure in either males or females. Stress-strain curves, passive distensibility and wall-lumen ratio of pressurized 4^th^ order mesenteric arteries are shown in Figure 3. Prenatal BPS exposure had no impact on mechanical properties of mesenteric arteries in either male or female offspring.

**TABLE 1:** EC_50_ values of mesenteric arteries isolated from BPS-exposed or control mice.

	Vehicle male		BPS male		Vehicle female		BPS female	
	LogEC_50_	CI	LogEC_50_	CI	LogEC_50_	CI	LogEC_50_	CI
Acetylcholine	−7.174	−7.552 to −6.889	−7.319	−7.621 to −7.019	−6.689	−7.032 to −6.351	−7.645^***^	−7.882 to −7.408
Phenylephrine	−6.437	−6.569 to −6.306	−6.538	−6.690 to −6.638	−6.476	−6.690 to −6.386	−6.715*	−6.859 to −6.605

*p < 0.05, ***p < 0.001 BPS vs. vehicle control. Confidence interval is expressed as 90% CI.

### Effect of sex and BPS on microvascular expression levels of eNOS and estrogen receptors

**FIGURE 4:**
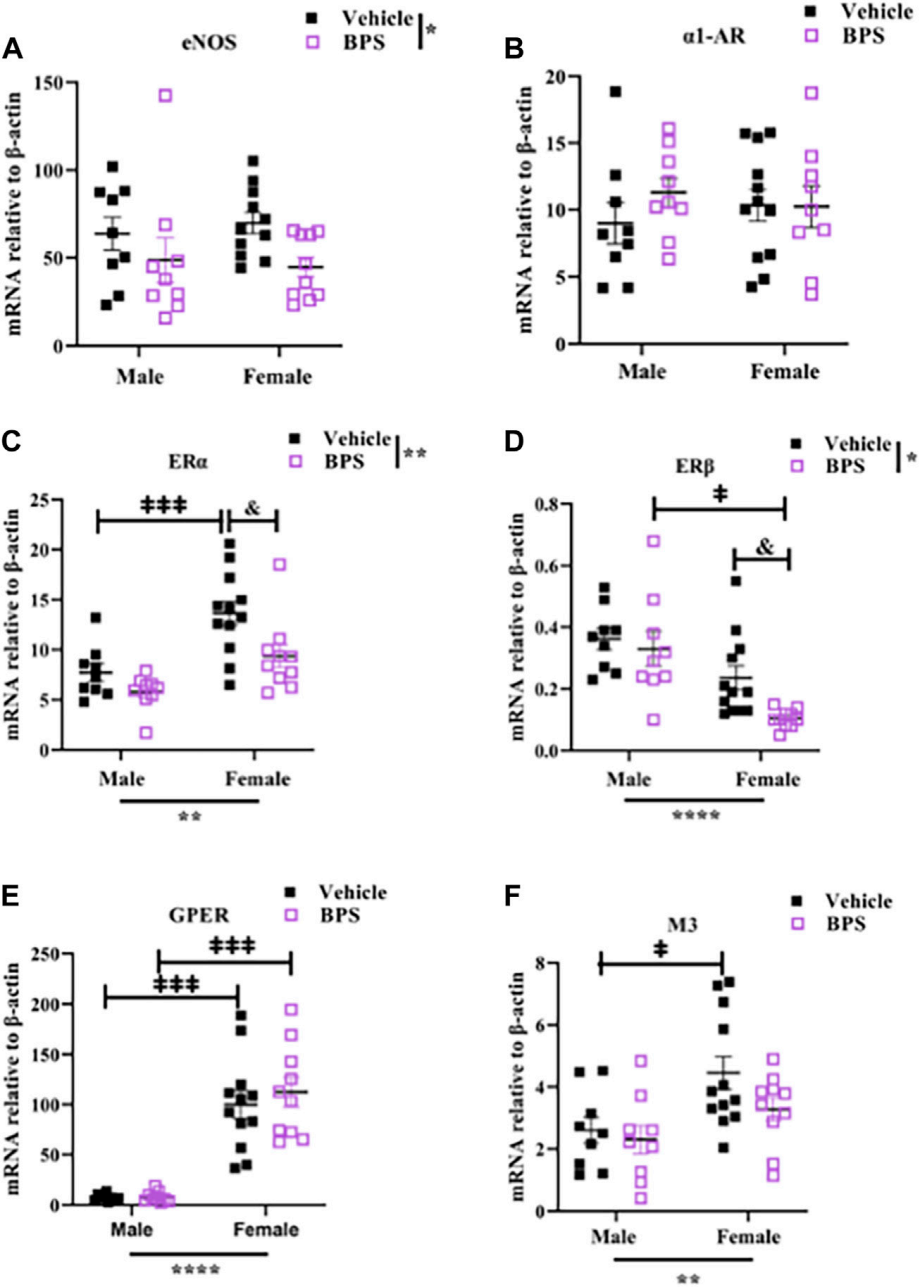
Gestational BPS exposure decreases mRNA expression of eNOS, ERα, and ERβ in microvessels. qRT-PCR was used to measure gene expression relative to β-actin in mesenteric vessels isolated from vehicle or BPS-exposed mice. **(A–F)** mRNA levels of eNOS, α1-AR (Phe receptor), ERα, ERβ, GPER and M3 (ACh receptor). Two-way ANOVA was used to determine the impact of treatment (BPS) or sex on gene expression. *p < 0.05; **p < 0.01 treatment or sex as a significant source of variation. & *p* < 0.05 BPS vs. vehicle by Sidak’s multiple comparison test. ‡*p* < 0.05; ‡‡‡*p* < 0.001; male vs. female by Sidak’s multiple comparison test.

Prenatal BPS exposure had a significant effect on the expression of eNOS, with a trend toward a decrease in both BPS-exposed male and female mesenteric arteries (Figure 4A). There was no influence of BPS on expression of α1-AR (Figure 4B). ERα was expressed at higher levels than ERβ, and sex was a significant determinant of both, with ERα higher in females and ERβ higher in males (Figures 4C,D). There was a significant decrease in the expression of ERα and ERβ in BPS-exposed vs. control female offspring (Figures 4C,D). While there were sex-specific differences in the expression of GPER and M3, prenatal BPS exposure had no impact on the expression of these receptors (Figures 4E,F).

### Inhibition of ER or eNOS abolishes the acute effect of BPS on relaxation in female vessels

**FIGURE 5:**
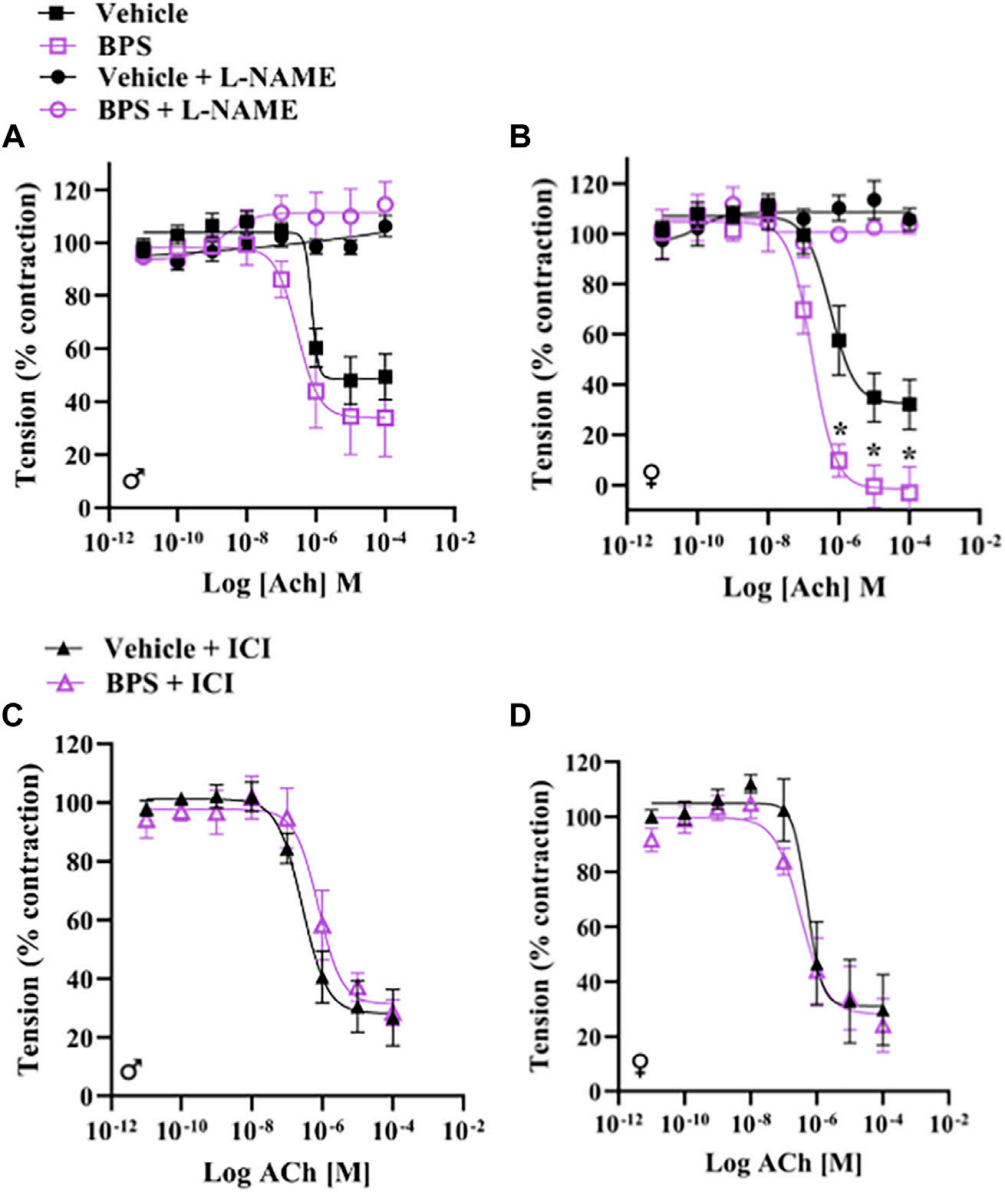
BPS-induced augmentation of dilation in female vessels is mediated by eNOS and ER. Aortic rings from male or female mice were mounted onto a wire myograph and exposed to BPS or vehicle for 30 min prior to generating concentration response curves to ACh. ACh-induced vasodilation was calculated as the % pre-contraction tension. Dilatory responses to ACh in the presence or absence of L-NAME in **(A)** male or **(B)** female aortic rings. ACh-induced dilatory responses in the presence of the ER antagonist, ICI, in **(C)** male or **(D)** female aortic rings. Curves were compared by Two-way ANOVA followed by Sidak’s post hoc test. *p < 0.05; **, *p* < 0.01; ***, *p* < 0.001 BPS vs. vehicle.

To determine if BPS has a direct effect on NO signaling, we incubated aortic rings in 250 nM BPS for 30 min prior to assessing contractile responses and Ach-induced dilation. Acute exposure to BPS markedly enhanced endothelium-dependent dilatory responses in female vessels (Figure 5B) but had no impact in male vessels (Figure 5A). The EC_50_ of the ACh response was also significantly decreased in females, suggesting an increase in sensitivity to ACh after acute exposure of the vessel to BPS (Table 2). The effect of acute BPS exposure on dilatory responses was endothelium dependent as there was no effect on responses to the NO donor, SNP. Further, BPS had no impact on contractile responses to Phe in either male or female vessels. Incubation of the vessels with L-NAME prior to adding Ach to the bath abolished differences in dilation between BPS and vehicle-treated (Figure 5B), suggesting that the effect of BPS on dilation is mediated by NO. Incubation with the ER antagonist, ICI 182,780, also abolished the effect of BPS on ACh-induced dilation in female vessels, and eliminated sex differences in dilation after BPS exposure (Figures 5C,D). These findings suggest that NO and ER signaling mediate the sex-specific effect of BPS on endothelium-dependent arterial relaxation.

**TABLE 2:** EC_50_ values of aortae acutely exposed to vehicle or BPS.

	Vehicle male		BPS male		Vehicle female		BPS female	
	LogEC_50_	CI	LogEC_50_	CI	LogEC_50_	CI	LogEC_50_	CI
Acetylcholine	−6.255	−6.502 to −6.028	−6.739	−7.066 to −6.477	−6.49	−6.593 to −6.231	−6.717****	−6.912 to −6.725
Phenylephrine	−5.772	−5.967 to −5.573	−5.766	−6.034 to −5.493	−5.745	−5.962 to −5.523	−5.712	−6.000 to −5.416

****p < 0.0001 BPS, vs. vehicle. Confidence interval is expressed as 90% CI.

### Prenatal BPS exposure does not influence glucose tolerance and insulin sensitivity

**FIGURE 6:**
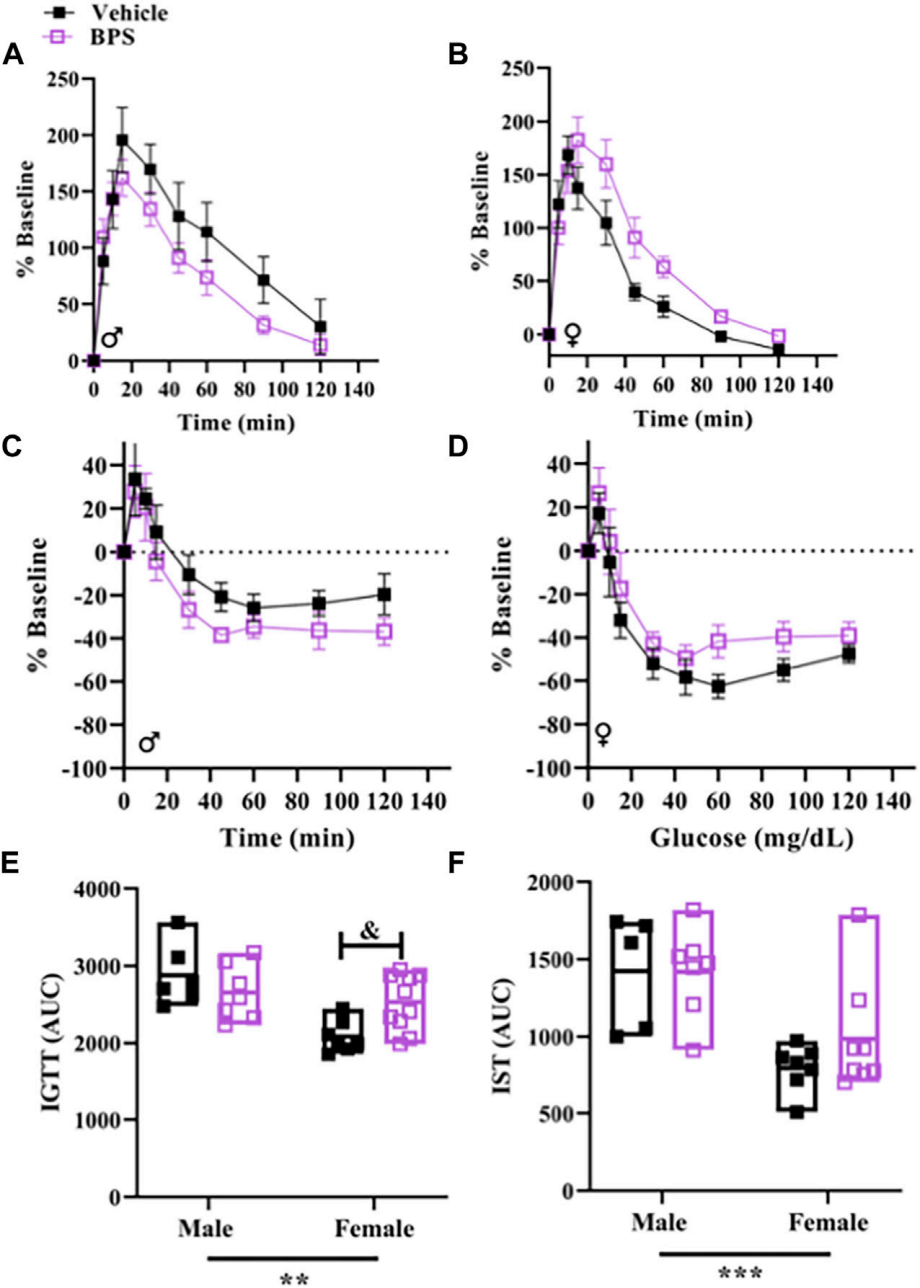
Glucose tolerance is impaired in female offspring born to BPS-exposed dams. Intraperitoneal glucose tolerance tests (IGTT) and insulin sensitivity tests (IST) were performed in adult offspring. **(A)** Serial glucose measurements in adult **(A)** male or **(B)** female mice challenged with glucose. Serial glucose levels in adult **(C)** male or **(D)** female mice challenged with insulin. **(D)** Serial glucose measurements in adult male mice challenged with insulin. Area under the curve (AUC) for **(E)** IGTT or **(F)** IST. AUC were compared by Two-way ANOVA with Sidak’s post hoc test. **p < 0.01; ***p < 0.001 sex as a significant source of variation. & *p* < 0.05 BPS vs. vehicle.

The IGTT and insulin sensitivity tests are shown in Supp Figure 6. Glucose tolerance and insulin sensitivity were both influenced by sex (Figures 6E,F). Area under the curve (AUC) of the IGTT was significantly higher in BPS-exposed females vs. control (Figures 6B,E), suggesting that BPS impaired glucose tolerance. In contrast, BPS exposure had no impact on glucose tolerance in males (Figures 6A,E). These findings show that the effect of BPS on later-life glucose handling is also sex-specific.

## Discussion

To our knowledge, this is the first study to assess vascular function after exposure to BPS during early life development. Pressure myography in intact microvessels revealed enhanced endothelium-dependent dilation in adult BPS-exposed females, while prenatal BPS exposure had no impact on male vessels. These findings suggest that BPS interferes with vascular development in exposed females and herein we provide evidence highlighting a role for eNOS activation by ER signaling as a potential mechanism that explains sex-specific vascular responses to BPS.

Studies investigating the impact of bisphenols on the vasculature have focused primarily on postnatal exposure and reveal wide-ranging effects on the cardiovascular system, leading to increased atherosclerosis risk and increased blood pressure in humans and rodent models (Gao and Wang, 2014; Saura et al., 2014). Exposure of 8-week-old mice to BPA was shown to induce eNOS uncoupling, leading to increased oxidative stress and hypertension (Saura et al., 2014). In agreement, recent findings (Easson et al., 2022) by our group demonstrate that postnatal exposure to the same dose of BPS used for prenatal exposure in the present study, leads to an impairment in microvascular endothelium-dependent dilation in males only, that is mediated by BPS-induced oxidative stress and eNOS uncoupling. Thus, BPS and other structural analogues may have opposing effects on endothelial function, such that increased NO production *via* interaction of BPS with ER counteracts the decrease in NO bioavailability due to BPS-induced oxidative stress. Our findings show that the modulation of NO production *via* interaction of BPS with ER signaling predominates in female vessels, providing a potential mechanism explaining sex-specific responses to BPS exposure. While postnatally exposed females were protected against BPS-induced impairments in endothelial function, the current findings show enhanced dilation in females when BPS exposure is restricted to the prenatal period. These findings suggest that ER may be involved in developmental establishment of NO-dependent dilatory responses in the microvasculature in a sex-dependent manner.

Sex differences in vascular function are largely dependent on estrogen, which is present in higher levels in females and influences vascular tone through the production of NO (Haynes et al., 2000; Hamilton et al., 2008; Knowlton and Lee, 2012). Estrogen can influence vascular function by binding to its receptors, ERα, ERβ, or GPER that are bound to the membrane where they activate rapid non-genomic signaling cascades or localized in the nucleus where they mediate genomic responses. In adulthood, ERα is the predominant isoform expressed in the vasculature, localized in the caveolae of endothelial cells where it complexes with eNOS to facilitate estrogen-dependent eNOS activation *via* the PI3K-Akt pathway using HSP90 as a chaperone protein (Haynes et al., 2000; Knowlton and Lee, 2012), whereas ERβ is primarily located in the nucleus of endothelial cells. ERβ has also been shown to activate NO production by eNOS, but only in embryonic cells rather than in adult vessels (Chambliss et al., 2002; Darblade et al., 2002; Knowlton and Lee, 2012). Data from the current study also show that ERα and GPER are the predominant isoforms expressed in the adult microvasculature and reveal sex differences with higher expression of ERα and GPER in female vessels, whereas male vessels expressed higher levels of ERβ compared to females.

ER activation has been shown to induce greater relaxation in female aortas compared to males and an intact endothelium was required to observe these sex differences in ER-mediated dilation (Kim et al., 2018) BPA and its substitutes including BPS are considered EDC with the ability to interact with hormone receptors and have been shown to exhibit estrogenic activity (Gao et al., 2015; Pelch et al., 2019). In the heart, bisphenols activate estrogen signaling, leading to changes in contractility that are mediated by eNOS (GillumPosnack et al., 2015; Ferguson et al., 2019). Wire myography experiments in the current study showed that sensitization of aortic rings to ACh-induced relaxation after acute exposure to BPS occurs only in female vessels and is prevented by inhibiting eNOS with L-NAME. Differences in endothelium-dependent vasodilation in vehicle and BPS-exposed vessels were also eliminated by an ER antagonist. These findings suggest that BPS activates non-genomic NO signaling *via* ER in a sex-dependent manner, suggesting this pathway to play a role in the sex-specific vascular effects observed in our mouse model of gestational BPS exposure. Since activation of genomic ER signaling can increase the expression of eNOS (MacRitchie et al., 1997), we examined the expression of eNOS and other receptors in mesenteric arteries of BPS-exposed mice to determine if changes in eNOS expression play a role in increased sensitivity to ACh. Our results reveal a significant effect of BPS on eNOS expression, with a trend towards decreasing expression in both male and female vessels. BPS exposure also influenced expression of both ERα and ERβ, with significant decreases in the expression of both in BPS-exposed vs. control female vessels. Therefore, changes in the expression of eNOS or ER isoforms do not explain the heightened dilatory responses observed in female mesenteric arteries but may be a compensatory response to increased eNOS activity. We cannot determine whether changes in vascular gene expression are a direct result of BPS exposure during vascular development or are secondary to perturbed vascular reactivity in postnatal life.

This study also found that the AGD index was significantly shortened in males. AGD is sexually dimorphic in mice, with males having a larger AGD than females. Anogenital distance is an indicator of reproductive tract masculinization and is thought to be a marker of androgen function *in utero*, with a longer AGD associated with increased androgen exposure, although estrogenic compounds have also been shown to influence AGD (Liu et al., 2014; Foster et al., 2006; Golub et al., 2010; Swan et al., 2015). Shortening of the AGD in male offspring has also been correlated with an increased risk for reproductive disorders (Schwartz et al., 2018). In the present study, both CRL and body weight were used to account for variations in size of the offspring, thus allowing us to observe true AGD feminization of the male offspring rather than shortening induced by overall stunted growth. The observed shortening is likely indicative of decreased androgen exposure in males *in utero,* suggesting both that BPS had a quantifiable effect on developmental hormone function and that this effect was sex dependent. This finding is in line with other observations on BPA exposure in humans, which have found decreased AGD in males after maternal BPA exposure (Mammadov et al., 2018; Sun et al., 2018). Prenatal BPA exposure in rodents has been shown to lead to AGD shortening in males (Murray et al., 2007; Christiansen et al., 2014). Other rodent studies using BPA, however, have also found no change or increased AGD (Gupta, 2000; Kobayashi et al., 2002; Golub et al., 2010). Variations in exposure timing, dosage, and route of administration therefore make drawing conclusions about the effects of BPA on AGD difficult. Our results specifically conflict with a previous study examining perinatal BPS exposure, which found that perinatal BPS exposure alone was not sufficient to induce shortening of AGD (Kolla et al., 2019). This difference may potentially be explained by the differences in timing of exposure, as the previous study began exposure at day 9 of pregnancy while exposure began at day 1 of pregnancy in the present report.

The current study found that prenatal BPS exposure impaired glucose uptake in female offspring only. This finding is in agreement with other studies in both humans and rodents showing insulin resistance and impaired glucose tolerance after bisphenol exposure (Callaghan et al., 2021). Impaired phosphorylation of the insulin receptor after BPA exposure (Batista et al., 2012) and reduced glucose transporter expression in response to BPS in mice have also been reported (Rezg et al., 2019). Exposing mice to BPA during pregnancy at a dose of 10 μg/kg/day, similar to the dose used in our study, was shown to impair glucose tolerance and increase insulin levels in both offspring and mothers, as well as alter maternal lipid profiles during gestation (Alonso-Magdalena et al., 2010). Our findings also support sex-specific effects of gestational bisphenol exposure on later-life glucose metabolism; however, we show that females are more vulnerable. Discrepancies in results of these two studies may be due to the use of BPS in the current study rather than BPA and our assessment of metabolic function at a younger age. Nevertheless, our data support previously published studies in human populations showing a positive association of urinary bisphenol levels with an increase in the risk of developing type 2 diabetes mellitus (Hwang et al., 2018). The programming effect of gestational BPS exposure on glucose metabolism does not explain changes in vascular function as hyperglycaemia is known to impair endothelial function.

In conclusion, this study found evidence that gestational exposure to a low dose of BPS programs heightened sensitivity to NO-mediated vasodilation in female offspring only. Further, we show that acute BPS exposure increased NO-mediated vasodilation in female vessels only, through an ER-dependent mechanism Together, these findings highlight the need to examine sex as a variable when studying the impact of EDC exposure and suggest that interaction of BPS with ER influences vascular function in a sex-dependent manner. (Reslan et al., 2013).

## Data Availability

The raw data supporting the conclusion of this article will be made available by the authors, without undue reservation.

## Supplementary material

Supplementary Figure 1 BPS exposure does not impact litter size or sex ratio.
