# Supplementary material for: Prenatal exposure to a low dose of BPS causes sex-dependent alterations to vascular endothelial function in adult offspring
Source: Front Toxicol. 2022 Oct 13;4:933572. doi: 10.3389/ftox.2022.933572 (PMC9606655; doi:10.3389/ftox.2022.933572)
Supplement: Supplementary file 1 [file Image1.pdf]

frontiers

Supplementary Material

1.1 Supplementary Figures

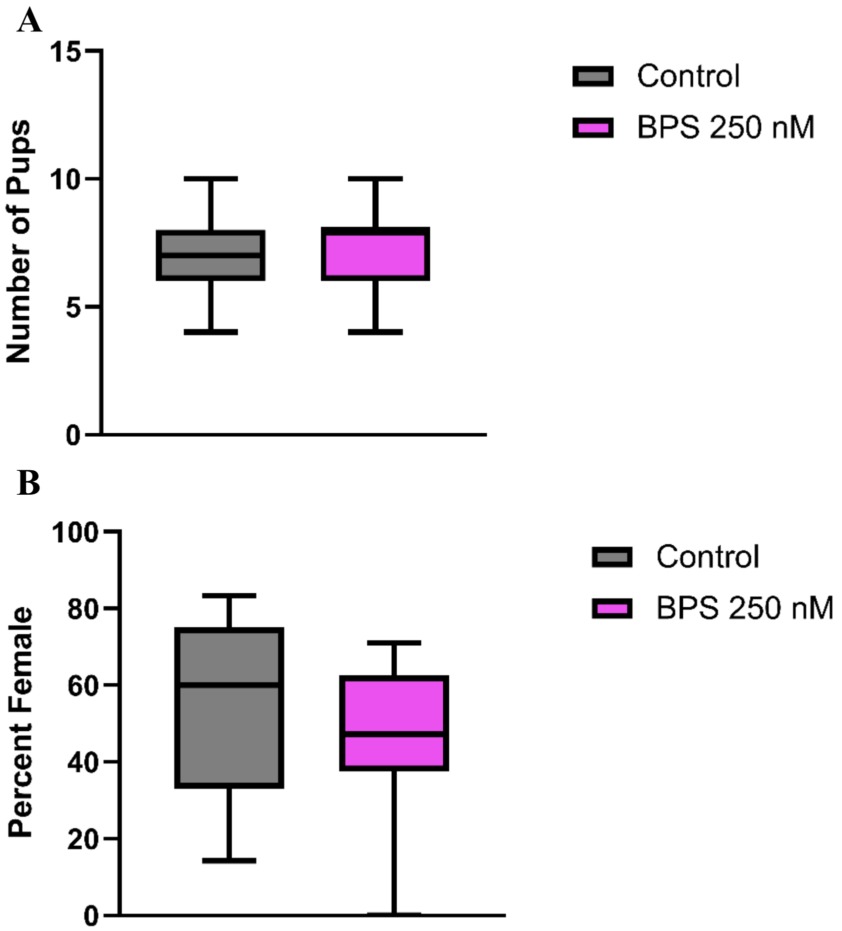

Supplementary Figure 1. A) Litter size and B) sex ratio of offspring born in each litter. Litters of mice were prenatally exposed to either BPS (n=20) or a vehicle control (n=24).
